# Supplementary material for: The Caribou (Rangifer tarandus) Genome
Source: Genes (Basel). 2019 Jul 17;10(7):540. doi: 10.3390/genes10070540 (PMC6678279; doi:10.3390/genes10070540)
Supplement: Supplementary file 1 [file genes-10-00540-s001.zip › Supplementary_Files/SupplementaryFileS2_Boveine_Reindeer_alignment.pdf]

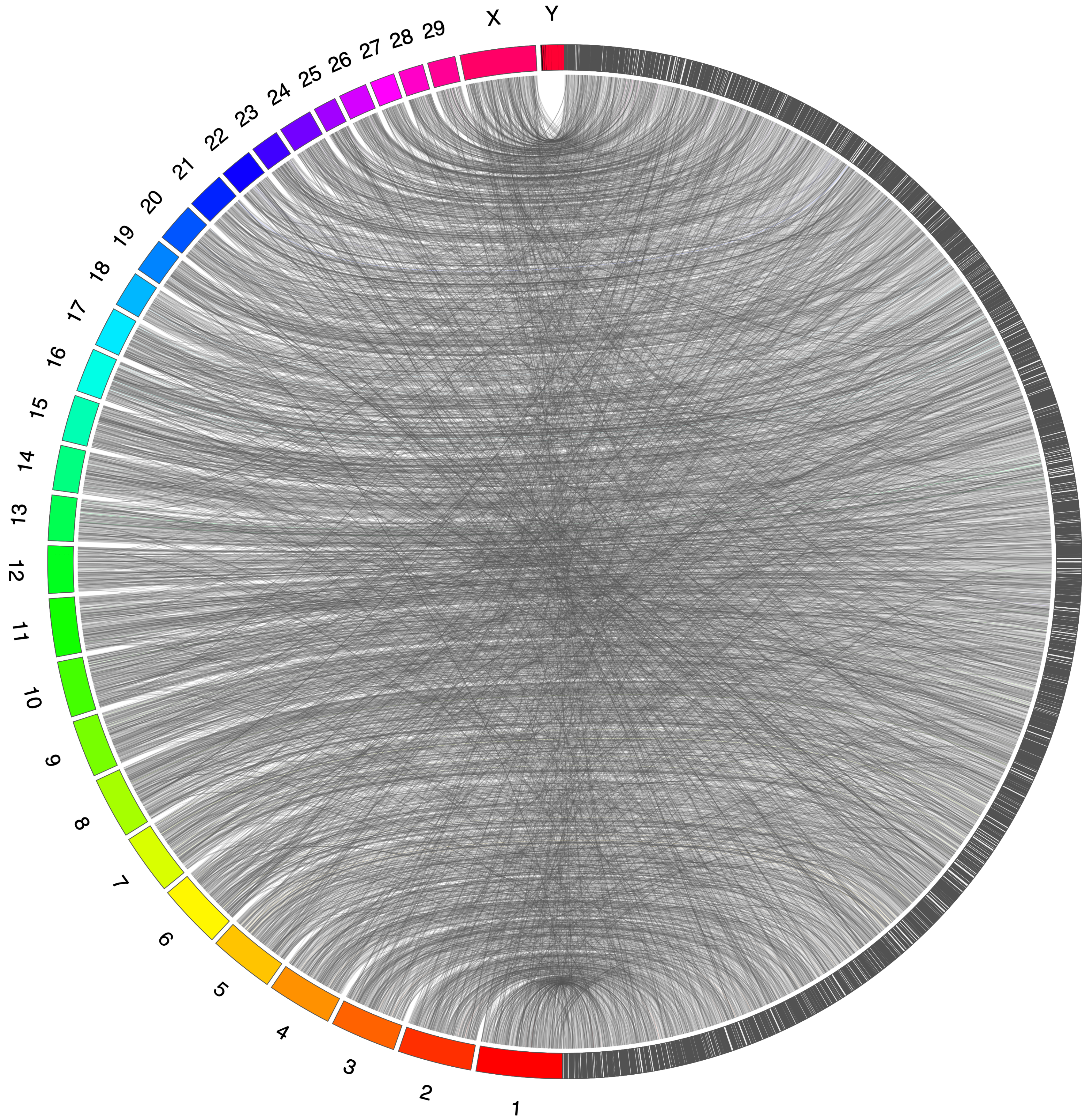

Supplementary File S2. A Jupiter plot showing an alignment between the bovine chromosomes and the reindeer genome assembly. The left of the circle shows the numbered bovine chromosomes, and the right of the circle has the largest 1,633 scaffolds from the reindeer assembly, which cover 75% of the bovine genome. Coloured bands represent synteny between the genomes, and lines crossing the circle indicate genomic rearrangements, or break points in the scaffolds.
